# Molecular Detection by Rolling Circle Amplification Combined with Deep Sequencing of Mixed Infection by Bovine Papillomaviruses 2 and 4 in Carcinoma In Situ of the Bovine Esophageal Mucosa

**DOI:** 10.3390/v16101558

**Published:** 2024-09-30

**Authors:** Bruna F. Matias, Michele Lunardi, Kátia C. B. Gonçalves, Laurival A. Vilas-Boas, Emanuele Gustani-Buss, Ana Paula F. R. L. Bracarense, Luiz Fernando C. Cunha Filho, Alice F. Alfieri, Amauri A. Alfieri

**Affiliations:** 1 Laboratory of Animal Virology, Department of Veterinary Preventive Medicine, Universidade Estadual de Londrina, Londrina 86057-970, Brazil; bruna_fonseka@hotmail.com (B.F.M.); michelelunardi@gmail.com (M.L.); aalfieri@uel.br (A.F.A.); 2 Multi-User Animal Health Laboratory, Molecular Biology Unit, Department of Veterinary Preventive Medicine, Universidade Estadual de Londrina, Londrina 86057-970, Brazil; 3 Post Graduate Program in Animal Health and Production, Department of Agrarian Sciences, University Pitagoras Unopar, Arapongas 86702-670, Brazil; vtluiz.cunha@gmail.com; 4 Department of General Biology, Universidade Estadual de Londrina, Londrina 86057-970, Brazil; kbrumatti@gmail.com (K.C.B.G.); lavboas@uel.br (L.A.V.-B.); 5 Department of Microbiology, Immunology and Transplantation, Rega Institute, KU Leuven-University of Leuven, Box 1030, 3000 Leuven, Belgium; 6 Laboratory of Animal Pathology, Department of Veterinary Preventive Medicine, Universidade Estadual de Londrina, Londrina 86057-970, Brazil; anapaula@uel.br; 7 National Institute of Science and Technology for Dairy Production Chain (INCT–LEITE), Universidade Estadual de Londrina, Londrina 86057-970, Brazil

**Keywords:** cattle, upper alimentary tract, esophagus, papilloma, histopathology, coinfection, polymerase chain reaction, next generation sequencing

## Abstract

Papillomaviruses (PVs) are oncogenic and infect the skin and mucosa of various host species. Considering the recent advances in research on PVs using rolling circle amplification (RCA) followed by high-throughput sequencing (HTS), in this study, we aimed to investigate the bovine papillomavirus (BPV) types associated with proliferative lesions in the upper alimentary tract of an affected bull and characterize the viral strains through complete genome sequencing using this strategy. We analyzed the PV strains associated with two hyperplastic esophageal lesions through PCR using degenerate primer pairs and RCA, followed by HTS. HTS of the libraries generated using RCA products provided the whole genome sequence of BPV4 present in squamous papilloma, whereas the complete genome sequence of BPV2 and subgenomic fragments of BPV4 were identified in carcinoma in situ (CIS). For the first time, we have sequenced BPV2 identified from the CIS of the bovine upper alimentary canal. Additionally, RCA followed by HTS allowed characterization of the mixed infection by BPV2 and BPV4 in this lesion. These data reveal that BPV4 is not the only BPV type present in CIS of the esophageal mucous membrane; moreover, a mixed infection caused by BPV2 and BPV4 at the tested anatomical site was demonstrated.

## 1. Introduction

Papillomaviruses (PVs) are small, non-enveloped oncogenic viruses with an icosahedral capsid and a genome composed of double-stranded circular DNA that is approximately 8000 bp in length [1,2]. PVs infect the skin and mucous membranes of various hosts, including humans. These infections usually cause benign lesions that spontaneously regress [3]. However, in some cases showing no occasional regression, malignant progression may occur, in which benign papilloma transforms into squamous cell carcinoma [4].

The papillomavirus genome contains open reading frames (ORFs) that encode the early (E) and late (L) proteins; E1 and E2 proteins promote viral replication, E4 participates in the production of virions, and the oncoproteins E5, E6, and E7 are linked to cellular transformation. The late proteins L1 and L2 are structural proteins involved in the formation of the capsid and the completion of the viral cycle [1,2].

Among the *Papillomaviridae* family, 44 types of bovine papillomavirus (BPV) have been reported to infect cattle. These diverse viruses belong to the genera *Deltapapillomavirus* (BPV1, 2, 13, and 14), *Dyoxipapillomavirus* (BPV7, 19, and 21), *Epsilonpapillomavirus* (BPV5 and 8), *Xipapillomavirus* (BPV3, 4, 6, 9, 10, 11, 12, 15, 17, and 23), unclassified *Dyokappapapillomavirus* (BPV16, 18, and 22), unclassified *Xipapillomavirus* (BPV20, 24, 26, 28, and 29), unclassified *Epsilonpapillomavirus* (BPV25), and unclassified types awaiting official classification (BPV 27, 30, 31, 32, 33, 34, 35, 36, 37, 38, 39, 40, 41, 42, 43, and 44) [5,6].

In cattle, BPVs can cause several clinical entities, such as cutaneous papillomatosis characterized by benign tumors at different anatomical sites on the skin, which can be histologically classified as fibropapillomas and true papillomas [7]. Other BPV infection-associated clinical conditions in cattle include bovine enzootic hematuria (BEH), a chronic inflammatory and neoplastic disease of the urinary bladder [4], and the development of tumors in the upper alimentary tract; both clinical alterations may be related to the chronic ingestion of bracken fern (*Pteridium aquilinum*). In addition to its toxic, carcinogenic, and mutagenic effects, *P. aquilinum* has an immunosuppressive effect, which contributes to papilloma persistence [8,9]. In tumors developed in the upper digestive tract, BPV4 has mainly been detected in squamous papillomas [10], whereas BPV2 is associated with fibropapillomas [11].

The rolling circle amplification (RCA) followed by high-throughput sequencing (HTS) supported significant advances in recent research on papillomaviruses. This untargeted amplification method has helped to sequence the genomes of existing and potential new viral types of PVs with greater sensitivity [12,13,14]. In this study, we aimed to investigate the BPV types associated with hyperplastic lesions located in the upper alimentary tract of an affected bull and to characterize the involved viral strains through complete genome sequencing using this molecular methodology.

## 2. Materials and Methods

### 2.1. Biological Samples and Histopathological Analysis

Through the post-slaughter inspection of the mucosa lining the whole upper alimentary tract, two proliferative lesions were identified and collected from the esophagus of a Girolando breed mature dairy bull provenient from a farm located in the North Central mesoregion of the Parana state, southern Brazil. A portion of each esophageal lesion was fixed by immersing in 10% neutral-buffered formalin and stained using hematoxylin and eosin for routine histopathological evaluation. The proliferative mucosal lesions were histopathologically evaluated as previously described [15]. The remaining fragment of each proliferative lesion was stored at −80 °C for further molecular evaluations.

The sample collection and all procedures performed in this study were based on ethical and animal welfare considerations. All the applicable international, national, and institutional guidelines for animal care and use were followed. This study was approved by the Ethics Committee for Animal Use at the Universidade Estadual de Londrina (Protocol No. 050.2021).

### 2.2. PCR Amplification of Partial BPV L1 and E1 Genes

Frozen fragments acquired from each esophageal lesion were disrupted and homogenized using TissueLyser LT (Qiagen, Hilden, Germany). Subsequently, total DNA was purified from these tissue homogenates using the DNeasy Blood and Tissue Kit (Qiagen, Hilden, Germany) following the instructions provided by the manufacturer. Purified DNA was stored at −80 °C for further molecular analyses. Aliquots of ultrapure sterile water were used as negative controls in all DNA extraction procedures.

To investigate the presence of PV strains potentially associated with the hyperplastic lesions collected from the esophageal mucosa of the dairy bull, three degenerate primer pairs designed to detect the conserved regions of the L1 and E1 genes of a broad spectrum of cutaneous and mucosal PV strains were tested using DNA purified from both lesions. The sequences and features of the primers used are listed in Table 1.

PCR mixtures (50 μL) contained 5 μL of the purified DNA, 0.4 to 1 μM of each primer, 200 μM of each deoxynucleoside triphosphate (dNTP) (Invitrogen, Carlsbad, CA, USA), 2.5 U of HotStar Taq DNA polymerase (Qiagen, Hilden, Germany), 1× PCR buffer, 1.5 to 2.5 mM MgCl_2_, and ultrapure sterile water used to make up the final volume. Amplification was performed with the following cycling profile: an initial step of 15 min at 95 °C, followed by 45 cycles of 1 min at 94 °C, 1 min at an optimal temperature for primer annealing (Table 1), and 1 min at 72 °C, with a final extension for 10 min at 72 °C. Aliquots of the PCR-amplified products were analyzed via electrophoresis using 2% agarose gels, stained with ethidium bromide (0.5 mg/mL), and examined under UV light.

### 2.3. Sequencing of Subgenomic Fragments

Amplified partial fragments of the BPV L1 and E1 genes were purified using a Wizard SV Gel and PCR Clean-Up System kit (Promega, Madison, WI, USA) following the protocol provided by the manufacturer. Next, direct sequencing was performed on a 3500 Genetic Analyzer (Applied Biosystems, Carlsbad, CA, USA) using the BigDye Terminator v.3.1 cycle sequencing kit (Applied Biosystems, Carlsbad, CA, USA) and the corresponding forward and reverse primers, according to the instructions provided by the manufacturer. The generated sequences were examined using the Phred application for quality analysis of chromatogram readings. The sequences with a base quality ≥20 were considered acceptable. The consensus sequences were determined using CAP3 software Version 3, and their identities were analyzed based on the public database GenBank using the BLASTn program.

### 2.4. Rolling-Circle Amplification

The BPV genomes were amplified using DNA purified from two esophageal lesions through the multiple-primed rolling-circle amplification (RCA) technique using the TempliPhi 100 amplification kit (Cytiva, Marlborough, MA, USA), following a previously reported protocol optimized for the amplification of papillomaviral genomes [19]. We verified the amplification of PV DNA through electrophoretic analysis of the RCA product (2 μL) using a 0.8% agarose gel, generating a band corresponding to multiple tandem copies of the full-length PV DNA.

The RCA products were purified using a Wizard SV Gel and PCR Clean-Up System kit (Promega, Madison, WI, USA) and quantified through fluorometry using a Qubit 4.0 (Invitrogen).

### 2.5. High-Throughput Sequencing (HTS)

Libraries were prepared using 1 ng of purified DNA using an Illumina DNA Library Prep kit (Illumina, San Diego, CA, USA). The Agilent D1000 ScreenTape kit (Agilent Technologies, Santa Clara, CA, USA) was used for the quality control of the library on the Agilent TapeStation 4150 system (Agilent Technologies, Santa Clara, CA, USA) and determination of the library size. The library was quantified on a Qubit 4.0 fluorometer using the Qubit dsDNA Quantification High Sensitivity Assay kit (Invitrogen, Waltham, MA, USA). The library was sequenced via the paired-end sequence strategy using the MiniSeq High Output kit (2 × 150 cycles) and MiniSeq system platform (Illumina, San Diego, CA, USA). Initially, the quality of the sequencing reads was evaluated using FastQC software version 0.11.3 (www.bioinformatics.babraham.ac.uk/projects/fastqc). To minimize possible base-calling errors, reads were trimmed based on a Phred quality score cutoff of 30; sequence reads shorter than 150 nucleotides (nt) were discarded. Complete BPV genomes were assembled using a *de novo* strategy using SPAdes software version 3.9.0 [20].

The reference-guided assembly of the sequencing reads obtained for the CIS was performed using the following steps. Firstly, MultiQC was used for quality control reporting [21], followed by Trimmomatic for trimming and filtering paired-end reads [22]. The BWA (Burrows–Wheeler Aligner) was employed for aligning reads to the BPV4 reference genome (accession number: X05817) [23], and SAMtools was used to process, sort, index, and calculate alignment statistics from BAM files [24]. The BCFtools package was employed to generate consensus sequences based on reference-guided assembly [25].

To annotate the genomes obtained from HTS, putative ORFs were identified using a combination of the ORF Finder program and BLASTp developed by the National Center for Biotechnology Information (NCBI) and compared with previously characterized BPV strains, while the theoretical isoelectric points and the molecular weights of the predicted viral proteins were estimated using the Expasy server (https://web.expasy.org/compute_pi/, accessed on 14 September 2024).

### 2.6. Phylogenetic Analysis

Pairwise and multiple sequence alignments at the nucleotide (nt) and amino acid (aa) levels and sequence similarities were estimated using ClustalW in the MEGA v11 software [26]. Complete nt sequences of the L1 gene derived from PV types, previously characterized through complete genome sequencing and representing the *Deltapapillomavirus* and *Xipapillomavirus* genera, were included in the analyses. Two phylogenetic trees were reconstructed based on the alignment of complete L1 nt sequences using the Maximum Likelihood method with the general time-reversible model in MEGA v11 software.

## 3. Results

**Figure 1:**
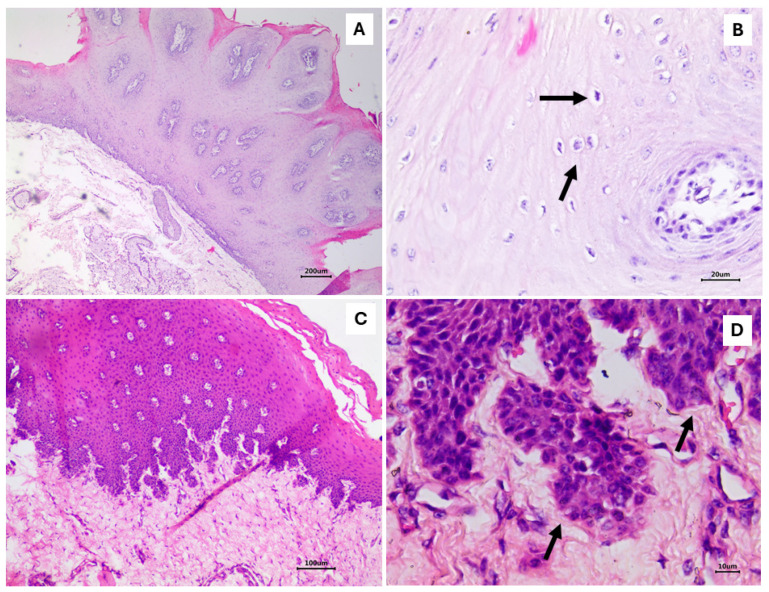
Esophageal mucosa of a Girolando dairy bull. (**A**) Marked epithelium proliferation and parakeratotic hyperkeratosis resulting in exophytic folding. Scale bar 200 μm; (**B**) acanthosis and koilocytosis (arrows). Scale bar 20 μm; (**C**) proliferation of disorganized epithelial cells without invasion through the basement membrane into the submucosa. Scale bar 100 μm; (**D**) epithelial proliferation without invasion of the basement membrane (arrows). Scale bar 10 μm. Figure (**A**–**D**)—hematoxylin–eosin.

Histopathological analyses revealed marked epithelial hyperplasia, parakeratotic hyperkeratosis, and koilocytosis, resulting in exophytic folding in the first lesion, which is characteristic of a squamous papilloma (Figure 1A,B). These cytopathic effects indicate BPV infection (Figure 1A,B). In the second lesion, the proliferation of disorganized and anaplastic epithelial cells without invasion through the basement membrane into the submucosa was detected, which characterizes a carcinoma in situ (CIS) and preneoplastic lesion (Figure 1C,D).

The selected PCR using the primer pairs FAP59/FAP64 and AR-E1F2/AR-E1R4, whose amplicons represented partial fragments of the BPV L1 and E1 genes, successfully yielded PCR products with the expected lengths for DNA samples from both lesions evaluated. In contrast, PCR with the MY primer pair resulted in the absence of amplification of either of the DNA samples. No amplicons were obtained from the negative controls included in the PCR assays. Direct sequencing of the FAP amplicon obtained from the squamous papilloma DNA sample generated a partial L1 nt sequence shown to belong to BPV4 by BLASTn evaluation (99.32% identity; accession number: OP682875). Moreover, the partial nt sequence of E1, which was obtained from the same lesion, showed the highest identity (98.96%) with a BPV4 strain available in GenBank (accession number: MW436424). Contrastingly, direct sequencing of the amplicon representing a partial fragment of the L1 gene obtained from the CIS lesion revealed the highest similarity of this amplicon with BPV2, with an identity of 99.78% (accession number: KF284153). Similarly, for the esophageal CIS DNA sample, sequencing of the PCR product obtained using the primer pair targeting the E1 gene generated a partial nt sequence highly similar to the same viral type, exhibiting 99.51% identity (accession number: MF045490) through BLASTn-based analysis.

Sequencing analysis of the library using the RCA product of the esophageal squamous papilloma generated a total of 1,796,770 reads. After trimming, 1,127,632 high-quality reads were retained for the sequence analysis. These sequences were *de novo* assembled into contigs that were further compared with the GenBank database using BLASTn and BLASTx. For the squamous papilloma-derived DNA sample, only one contig was characterized as PV, representing the complete genome sequence of BPV4 (7274 nt, GC content of 41.9%, mean coverage of 4081, 504,788 used reads, and 40,038 unused reads). Through BLASTn analysis, the highest identity obtained by comparison with other BPV complete genomic sequences deposited in GenBank was 99.11% with the BPV4 strain 4827RS16-BR node 9 recovered from a teat papilloma in cattle in Brazil in 2016 (accession number: MW436424).

In contrast, the library obtained from the RCA product amplified from the CIS lesion generated a total of 1,867,724 reads, with 1,310,390 high-quality reads remaining after trimming. From this esophageal lesion, *de novo* assembly generated one contig representing the whole-genome sequence of the BPV2 strain (7947 nt, GC content of 45.8%, mean coverage of 2177, 266,312 used reads, and 388,883 unused reads). BLASTn analysis of this sequence revealed that the BPV2 strains E170428-1 and E181025 (accession numbers: LC510376 and LC510384), identified from equine sarcoids in Japan in 2017 and 2018, respectively, exhibited the closest similarity to the BPV2 genome obtained (nt identity of 99.94%). Additionally, *de novo* assembly generated three other contigs corresponding to BPV subgenomic sequences. These sequences were highly similar to BPV4 and represented partial fragments of the L1 (223 nt, mean coverage of 0.946, and 2 used reads), L2 (334 nt, mean coverage of 1.799, and 7 used reads), and E7 (208 nt, mean coverage of 0.878, and 2 used reads) genes (Table 2). When compared with the BPV4 genome sequenced from the esophageal squamous papilloma, the partial nt sequences of the L1, L2, and E7 genes generated from the CIS by *de novo* assembly exhibited identities of 100, 99.7, and 100%, respectively.

Through the reference-guided assembly employing the BPV4 reference genome, the reads obtained from the CIS lesion generated 12 different contigs that mapped to most genes of the BPV4. These nucleotide sequences represented partial fragments of the E7 (263 nt, mean coverage of 99.619, 2 used reads, and 1,817,830 unused reads), E1 (194 nt, mean coverage of 100, and 4 used reads; 913 nt, mean coverage of 100, and 10 used reads; 149 nt, mean coverage of 100, and 1 used read), E2 (147 nt, mean coverage of 100, and 1 used read; 341 nt, mean coverage of 100, and 4 used reads), L2 (627 nt, mean coverage of 100, and 11 used reads; 148 nt, mean coverage of 100, and 1 used read), and L1 (216 nt, mean coverage of 100, 2 used reads; 147 nt, mean coverage of 94.23, and 1 used read; 519 nt, mean coverage of 100, and 8 used reads; 166 nt, mean coverage of 100, and 3 used reads) genes. The comparison of these CIS BPV4 partial sequences with the BPV4 strain sequenced from the squamous papilloma revealed identities varying from 99.4 to 100%.

**Figure 2:**
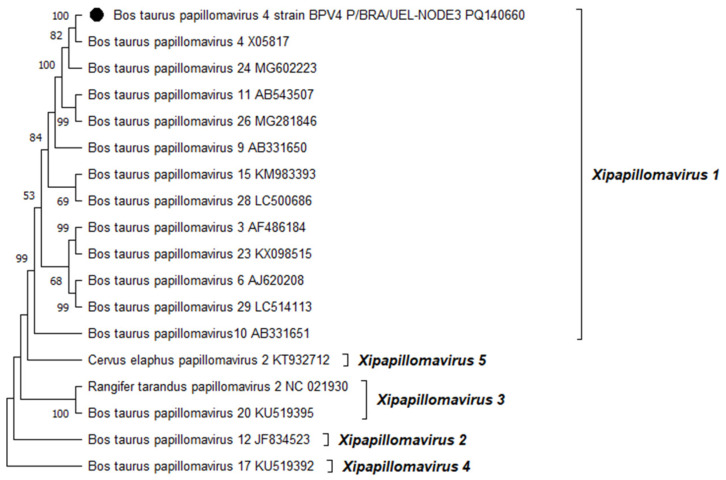
Phylogenetic analysis of the Brazilian bovine papillomavirus strain identified in a squamous papilloma developed in the esophagus of a dairy bull from southern Brazil using complete L1 gene nucleotide sequences. The evolutionary history was inferred using the maximum likelihood method and the general time reversible model. The tree with the highest log likelihood (−18,441.70) is shown. Numbers at internal nodes represent the bootstrap support values (percentages) determined for 1000 replications (values < 50 are not shown). The tree is drawn to scale, with branch lengths measured in the number of substitutions per site. We included 18 nucleotide sequences in this analysis. There were a total of 1580 positions in the final dataset. Evolutionary analyses were conducted using MEGA11 [26]. The Brazilian bovine papillomavirus strain sequenced in this study is indicated using a black-filled circle. Representatives of the viral PV types belonging to the five species of the genus *Xipapillomavirus*, recognized by the International Committee on Taxonomy of Viruses (ICTV), are shown. The GenBank accession numbers of the PV types included in the phylogenetic analysis are indicated.

**Figure 3:**
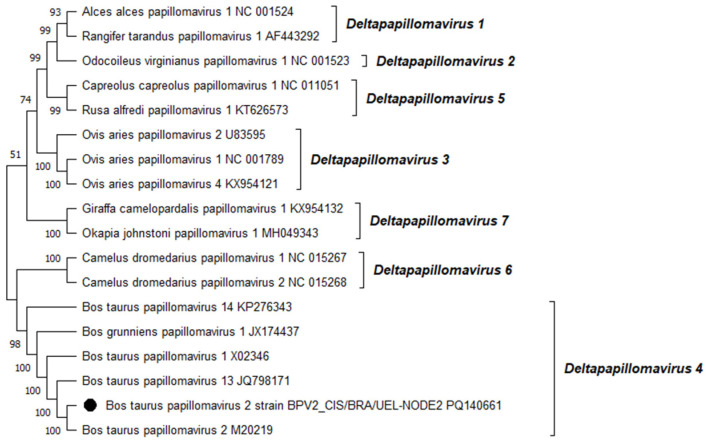
Phylogenetic analysis of the Brazilian bovine papillomavirus strain identified in a carcinoma in situ lesion developed in the esophagus of a dairy bull from southern Brazil using complete L1 gene nucleotide sequences. The evolutionary history was inferred using the maximum likelihood method and the general time reversible model. The tree with the highest log likelihood (−17,206.33) is presented. Numbers at internal nodes represent the bootstrap support values (percentages) determined for 1000 replications (values < 50 are not shown). The tree is drawn to scale, with branch lengths measured in the number of substitutions per site. This analysis involved 18 nucleotide sequences; the final dataset comprised a total of 1537 positions. Evolutionary analyses were conducted in MEGA11 [26]. The Brazilian bovine papillomavirus strain sequenced in this study is represented by a black-filled circle. Representatives of the viral PV types belonging to the seven species of the genus *Deltapapillomavirus*, recognized by the International Committee on Taxonomy of Viruses (ICTV), are shown. The GenBank accession numbers of the PV types included in the phylogenetic analysis are indicated.

Phylogenetic reconstruction based on the complete L1 nt sequences of all PVs, already characterized through the complete genome sequencing and classified in the *Xipapillomavirus* genus, confirmed that the BPV4 genome derived from esophageal squamous papilloma belongs to the *Xipapillomavirus 1* viral species (Figure 2). Additionally, the phylogenetic tree of the complete L1 nt sequences of PVs fully sequenced and classified in the *Deltapapillomavirus* genus revealed that the Brazilian BPV2 strain present in the CIS lesion on the same organ belonged to the *Deltapapillomavirus 4* viral species, with high statistical support (Figure 3).

The complete genomic sequence of the BPV4 strain characterized from the esophageal squamous papilloma was deposited in the GenBank database under the accession number PQ140660. The whole genome of the BPV2 strain sequenced from the esophageal CIS lesion has been submitted under the accession number PQ140661, while the nt sequences obtained from the CSI lesion by the *de novo* assembly, representing partial fragments of the BPV4 L2, E7, and L1 genes, were deposited under the accession numbers PQ140662 to PQ140664, respectively. In addition, the BPV4 subgenomic sequences generated by the reference-guided assembly of the CIS lesion were deposited under the accession numbers PQ365677 to PQ365688. Summary data of the predicted proteins identified in the BPV4 and BPV2 complete genomes through the ORF Finder program are presented in Table 3.

## 4. Discussion

In this study, BPV2 and BPV4 were identified in lesions with different histological classifications developed in the esophagus of an asymptomatic dairy bull in southern Brazil. The viral types were detected by PCR assays using degenerate primers that amplify a broad range of PV sequences and through an untargeted amplification strategy involving the RCA of BPV genomes followed by sequencing via HTS. Additionally, deep sequencing of the RCA product derived from the esophageal CIS lesion revealed mixed infection by BPV2 and 4; the BPV2 complete genomic sequence and partial nucleotide sequences from most genes of the BPV4 were obtained.

Although BPV4 is the viral type mostly associated with tumor lesions that develop in the mucous membrane lining the upper digestive canal of cattle, both BPV4 belonging to the genus *Xipapillomavirus* and BPV2 belonging to the genus *Deltapapillomavirus* serve as causal agents of hyperplastic lesions of the bovine upper alimentary tract, as previously reported [10,11,27]. The BPV4 infection was associated with progression to malignancy [28,29]. However, BPV2 infections seem to rarely lead to fibropapillomas at this anatomical site [11]. As only BPV4 was identified in alimentary cancers of cattle, a link between BPV2 infection of the upper digestive tract and the development of malignancy has not been established in cattle [11].

In this study, the BPV2 complete genomic sequence was amplified from an esophageal CIS DNA sample and sequenced. To the best of our knowledge, we have identified this viral type in a premalignant lesion of the bovine upper alimentary tract for the first time. Additionally, the results reflect that a mixed infection with BPV4 can be characterized in the CIS lesion through RCA amplification followed by HTS. A recent investigation carried out in the midwestern region of Brazil revealed the occurrence of a mixed infection caused by BPV4 and BPV2 in a squamous papilloma developed in the esophagus of a dairy cow. Viral types were identified using conventional PCR employing the degenerate primer pairs FAP59/FAP64 and AR-E1F2/AR-E1R4, followed by cloning and sequencing of the amplified L1 and E1 gene fragments [30]. Mixed infection by BPV2 and BPV4 has been previously described in a squamous papilloma of the esophagus of a dairy cow; however, for the first time, this study presents its occurrence in a CIS in the same organ of cattle.

The RCA and HTS of the complete genome of BPV4 in the squamous papilloma of the esophagus of the dairy bull are comparable to the previous findings. Previously, BPV4 was reported in papillomatous lesions without malignant progression developed in cattle raised in Scotland and England. The conventional PCR-based molecular analyses of these biological samples confirmed the viral type, whereas histopathological analyses revealed papillomatous changes throughout the upper alimentary tract [31]. In Italy, through histopathological evaluation, esophageal papilloma (inverted and exophytic) was detected in 147 cattle among a total of 1133 animals slaughtered between 2000 and 2001; BPV4 was present in >60% of the examined samples [32].

In addition to the viral types commonly detected in tumors in the upper digestive tract, studies carried out in India revealed BPV5 in lesions from this anatomical site of cattle for the first time. This viral type was detected in six hyperproliferative lesions acquired from the rumen, reticulum, and esophagus of cattle and was histologically diagnosed to be fibropapillomas and papillomas [33]. In the present study, we did not detect BPV5 in the lesions developed in the upper alimentary tract of the dairy bull via broad-spectrum PCR assays using consensus primers or untargeted amplification by RCA, combined with deep sequencing.

Although BPV4 was associated with the emergence of papillomas in the upper alimentary tract of cattle, a retrospective survey was conducted using 47 papillomas in the mouth and esophagus of 30 animals with upper digestive canal carcinomas, collected from southern Brazil between 2003 and 2014; PCR assays conducted using both the FAP primer pair and specific primers targeting the BPV4 L1 gene did not reveal BPV DNA in these lesions [34]. In the present study, RCA of the BPV genomes followed by HTS successfully identified BPV2 and 4 sequences in the CIS lesion, as well as BPV4 in a squamous papilloma in the upper alimentary tract of a dairy bull, which indicates that BPV4 is not the only BPV type present in the CIS developed in the bovine esophageal mucous membrane. This molecular strategy potentially allows the identification of diverse BPV types, even in mixed infections, in different hyperproliferative lesions of cattle, deepening the research on BPV. In the northern region of Brazil, cutaneous papillomas collected from cattle were reported to contain three viral types previously characterized in one sample and BPV24, a novel type of BPV, in another sample; notably, both samples were collected from the skin lesions of a single animal [35]. In an investigation carried out on cows slaughtered in another state of southern Brazil, RCA- and HTS-based assays using 23 teat warts revealed seven known viral types (BPV3, 4, 6, 8, 9, 12, and 27); moreover, 14 new types (BPV30–BPV43) were characterized for the first time [14].

In conclusion, the present study, for the first time, demonstrates the association of BPV2 with a CIS lesion in the bovine upper alimentary tract and reveals the occurrence of mixed infection by BPV2 and BPV4 in the tumor. Since mixed infection in esophageal CIS could not be detected by common molecular tools, such as PCR using degenerate primers followed by Sanger sequencing of the amplicons, the use of untargeted molecular techniques, such as RCA and HTS, is essential for advancement in PV research. This is the first study that revealed the association between BPV and proliferative lesions in the bovine upper digestive canal using this molecular strategy.

## Figures and Tables

**Table 1:** Sequences and features of degenerate primers used for the PCR amplification of partial papillomavirus genes.

Primer	Genomic Region Target	Polarity	Sequence (5′-3′) ^a^	Expected Amplicon Length (bp)	Annealing Temperature
MY 11 ^b^	L1	Forward	GCMCAGGGWCATAAYAATGG	450	55 °C
MY 09 ^b^	L1	Reverse	CGTCCMARRGGAWACTGATC		
FAP59 ^c^	L1	Forward	TAACWGTNGGNCAYCCWTATT	480	50 °C
FAP64 ^c^	L1	Reverse	CCWATATCWVHCATNTCNCCATC		
AR-E1F2 ^d^	E1	Forward	ATGGTNCAGTGGGCNTATGA	552	50 °C
AR-E1R4 ^d^	E1	Reverse	ATTNCCATCHADDGCATTTCT		

^a^ Degenerate nucleotides: M = A or C; R = A or G; W = A or T; Y = C or T; V = A, C or G; H = A, T or C; N = A, G, C or T; D = A, T or G. ^b^ [16]; ^c^ [17]; ^d^ [18].

**Table 2:** Types of bovine papillomavirus identified in esophageal lesions with distinct histopathological classifications using different amplification and sequencing strategies.

Histological Type	Amplification and Sequencing Methods			
	PCR/Sanger Sequencing		RCA/High Throughput Sequencing	
	Partial L1 Gene ^a^	Partial E1 Gene ^b^	Complete Genome	Partial BPV Genes
Squamous papilloma	BPV4	BPV4	BPV4 ^c^	-
Carcinoma in situ	BPV2	BPV2	BPV2 ^d^	BPV4

Primers: ^a^ FAP; ^b^ AR-E1F2/AR-E1R4. GenBank accession Nos.: ^c^ PQ140660; ^d^ PQ140661.

**Table 3:** Features of the predictive ORFs identified in BPV genomes sequenced from bovine esophageal lesions through high-throughput sequencing of rolling circle amplification products.

BPV Strain	ORF	ORF Location	Length (nt)	Length (aa)	Molecular Mass (KDa)	pI
**BPV4 ^a^**	E5	333–461	129	42	4.995	4.21
	E8	448–585	138	45	5.312	3.58
	E7	716–1012	297	98	10.993	4.41
	E1	1002–2831	1830	609	69.506	5.98
	E2	2773–4005	1233	410	46.416	9.96
	E4	3299–3739	441	146	16.728	6.31
	L2	4020–5597	1578	525	57.244	4.84
	L1	5608–7128	1521	506	57.575	7.14
**BPV2 ^b^**	E6	935–1348	414	137	15.867	8.97
	E7	1323–1706	384	127	13.601	9.08
	E1	1693–3513	1821	606	68.043	6.10
	E8	2048–2278	231	76	8.710	9.88
	E2	3455–4693	1239	412	45.681	6.15
	E4	4038–4379	342	113	12.688	10.75
	E5	4735–4869	135	44	5.210	4.35
	L2	5038–6441	1404	467	49.433	4.94
	L1	6454–7947	1494	497	55.610	8.44

^a^ GenBank accession No.: PQ140660. ^b^ GenBank accession No.: PQ140661.

## Data Availability

The original data presented in the study are openly available in GenBank database under the following accession numbers: PQ140660-PQ140664; PQ365677 to PQ365688.
